# Development of a Multiplexed Bead-Based Suspension Array for the Detection and Discrimination of *Pospiviroid* Plant Pathogens

**DOI:** 10.1371/journal.pone.0084743

**Published:** 2014-01-03

**Authors:** Sharon L. van Brunschot, Jan H. W. Bergervoet, Daniel E. Pagendam, Marjanne de Weerdt, Andrew D. W. Geering, André Drenth, René A. A. van der Vlugt

**Affiliations:** 1 Plant Biosecurity Cooperative Research Centre, Bruce, Australian Capital Territory, Australia; 2 School of Agriculture and Food Sciences, The University of Queensland, St Lucia, Queensland, Australia; 3 Plant Research International, Wageningen University and Research Centre, Wageningen, The Netherlands; 4 Commonwealth Scientific and Industrial Research Organisation Mathematics, Informatics and Statistics, Dutton Park, Queensland, Australia; 5 Centre for Plant Science, The University of Queensland, St Lucia, Queensland, Australia; Deutsches Krebsforschungszentrum, Germany

## Abstract

Efficient and reliable diagnostic tools for the routine indexing and certification of clean propagating material are essential for the management of pospiviroid diseases in horticultural crops. This study describes the development of a true multiplexed diagnostic method for the detection and identification of all nine currently recognized pospiviroid species in one assay using Luminex bead-based suspension array technology. In addition, a new data-driven, statistical method is presented for establishing thresholds for positivity for individual assays within multiplexed arrays. When applied to the multiplexed array data generated in this study, the new method was shown to have better control of false positives and false negative results than two other commonly used approaches for setting thresholds. The 11-plex Luminex MagPlex-TAG pospiviroid array described here has a unique hierarchical assay design, incorporating a near-universal assay in addition to nine species-specific assays, and a co-amplified plant internal control assay for quality assurance purposes. All assays of the multiplexed array were shown to be 100% specific, sensitive and reproducible. The multiplexed array described herein is robust, easy to use, displays unambiguous results and has strong potential for use in routine pospiviroid indexing to improve disease management strategies.

## Introduction

Viroids are the smallest plant pathogens known to date. They are autonomously replicating, unencapsidated infectious RNAs that move systemically throughout an infected plant. Their circular, single-stranded genomes range in size from ∼250–400 nucleotides (nt), and do not encode any proteins [1], [2]. All of the known viroid species are classified into two families, *Pospiviroidae* and *Avsunviroidae*, containing five and three genera, respectively [3]. The genus *Pospiviroid* (family *Pospiviroidae*) contains ten recognized species: *Chrysanthemum stunt viroid* (CSVd), *Citrus exocortis viroid* (CEVd), *Columnea latent viroid* (CLVd), *Iresine viroid 1* (IrVd-1), *Mexican papita viroid* (MPVd), *Pepper chat fruit viroid* (PCFVd), *Potato spindle tuber viroid* (PSTVd), *Tomato apical stunt viroid* (TASVd), *Tomato chlorotic dwarf viroid* (TCDVd), and *Tomato planta macho viroid* (TPMVd) [3]. Recent research has demonstrated the conspecificity of MPVd and TPMVd, and accordingly their reclassification to a single species, namely TPMVd, has been proposed [4].

Pospiviroids are responsible for economically important diseases of horticultural and agricultural crops, including tomato (*Solanum lycopersicum*), potato (*Solanum tuberosum*), pepper (*Capsicum annuum*), citrus (*Citrus* spp.) and ornamental chrysanthemum (*Chrysanthemum morifolium*). All pospiviroid species, except IrVd-1, are able to infect tomatoes and produce similar symptoms such as stunting, epinasty, leaf distortion, smaller fruit and reduced yield [5]–[10]. In the past two decades, severe outbreaks of pospiviroid diseases have been identified in cultivated tomato and pepper in various countries including Australia, China, Israel, Japan, New Zealand, North and South America, and several countries in the European Union (EU) [6], [10]–[18].

Pospiviroids are now the subject of increased phytosanitary concern worldwide. Currently, PSTVd and CSVd (in planting material of *C. morifolium*) have an explicit quarantine pest status in the EU (EU Plant Health Directive 200/29/EC), and accordingly member states have a statutory obligation to control these viroids. Costly outbreaks of CEVd, CLVd and TASVd in protected tomato cropping have prompted recommendations for the revision of their phytosanitary status in the EU [15], [19]–[21]. For all countries, maintaining a pest-free status for the continued exclusion of PSTVd and other pospiviroids is of great economic importance for the preservation of open trade.

The origins of pospiviroid outbreaks are often unknown. Primary inoculum sources have previously been linked to infected planting material, including infected seed lots, and also to asymptomatic viroid-infected ornamental plants [22]–[24]. Accordingly, the key method of control and management of pospiviroid diseases relies on the indexing of planting material and asymptomatic ornamental hosts as part of quarantine and certification schemes to prevent the introduction and spread of the pathogen. For routine indexing, conventional RT-PCR methods using degenerate primers are commonly used [6], [25], [26]. More recently, real-time RT-PCR methods have become available for the detection of several pospiviroids [21], [27]–[29]. To date, no multiplexed assays for the identification and differentiation of all nine species of pospiviroid have been developed. Significant improvements in the efficiency of pospiviroid detection could be achieved by using multiplexed detection methods, enabling faster response to incursion events and improved biosecurity outcomes.

Multiplexing technologies that enable the simultaneous detection of multiple nucleic acid sequences in a single reaction can greatly reduce the time, cost and labor associated with conventional single reaction detection technologies. The Luminex MagPlex-TAG microsphere system is a recently developed platform for multiplexed nucleic acid detection. This technology has proven its value for the multiplexed detection of pathogens in clinical settings [30]–[32]. This system incorporates 6.5 µm carboxylated, superparamagnetic polystyrene microspheres that are internally labeled with a spectrally distinct fluorescent dye and pre-coupled with an anti-MagPlex-TAG oligonucleotide sequence. Different microsphere sets can be distinguished by their spectral addresses, and when combined, up to 150 different nucleic acid sequence targets can be simultaneously detected in a single reaction.

The experimental approach for the Luminex MagPlex-TAG microsphere system involves a generic multiplexed RT-PCR step, followed by a multiplexed asymmetric PCR step termed Target Specific Primer Extension (TSPE). In this step, a primer internal to the multiplexed amplification product will hybridize, and be extended, only when there is a sequence match. Resultant TSPE products are biotinylated and labeled with complementary MagPlex-TAG sequences at their 5′ end. TSPE products are then hybridized to the MagPlex-TAG microsphere mixture (MagPlex-TAG/anti-MagPlex-TAG hybridization), and a fluorescent reporter molecule is used to detect incorporated biotin. The bead-TSPE product complexes are then detected on the Luminex instrument.

To determine if a sample is positive or negative in a particular assay within an array, a threshold for positivity (also referred to as the cut-off value) is required. Most studies describing Luminex bead-based arrays for nucleic acid detection define the threshold for positivity for all assays within an array as a value that exceeds that of the background (noise) by an arbitrary value or factor (e.g. see citations [33]–[38]). Such broad and subjective approaches may not account for the unique properties of each assay, and the complex interplay between the individual assays of the multiplexed array. These methods lack a suitable statistical assessment of the variability exhibited in median fluorescence intensity (MFI) values and therefore suffer from poor statistical rigor. Moreover, an arbitrarily defined threshold for positivity has no objective statistical meaning and provides no information of the percentage of false positives attributed to samples analyzed using that method [39].

Here we describe the development and validation of a true multiplex PCR-Luminex MagPlex-TAG bead suspension array for the generic and individual detection of all nine currently recognized species in the genus *Pospiviroid*. This 11-plex array has a unique hierarchical assay design, with the incorporation of a near-universal assay to detect all pospiviroid species (except CLVd). An internal control assay for the co-amplification of the plant mRNA, targeting the NADH dehydrogenase subunit 5 (*nad5*) gene, was included for quality assurance purposes to exclude false negative results. In addition, we also describe the development of a statistically rigorous method for setting thresholds for positivity for the analysis of multiplexed bead-based arrays.

## Results

### Development of the Multiplexed Bead-based Array

Due to the high level of sequence divergence displayed between CLVd and the remaining eight species within the genus *Pospiviroid* (∼50–70% nt sequence identity), an entirely universal assay could not be designed. Instead, one near-universal assay to detect eight pospiviroid species (excluding CLVd), nine species-specific assays and one plant internal control assay were designed to be tested simultaneously in the multiplexed bead array, enabling the detection and discrimination of all nine pospiviroids in a single, internally controlled assay. An *in silico* analysis (Table 1) demonstrated that the majority of the currently recognized sequence-characterized isolates of each pospiviroid species should theoretically be correctly identified using the multiplexed array described in this study.

**Table 1:** Predictions of the number of isolates of each pospiviroid species that will be detected using universal and species-specific assays of the Luminex MagPlex-TAG pospiviroid array.

Viroid species	Acronym	Number ofGenBanksequences^b^	Number of isolates predicted to be detected with each assayc,d									
			CSVd	CEVd	CLVd	IrVd-1	PCFVd	PSTVd	TASVd	TCDVd	TPMVd	PospUni
*Chrysanthemum stunt viroid*	CSVd	50	50									48
*Citrus exocortis viroid*	CEVd	204		204								200
*Columnea latent viroid*	CLVd	74			71							N/A
*Iresine viroid 1*	IrVd-1	7				7						7
*Pepper chat fruit viroid*	PCFVd	38					38					38
*Potato spindle tuber viroid*	PSTVd	225						218				222
*Tomato apical stunt viroid*	TASVd	15							14			15
*Tomato chlorotic dwarf viroid*	TCDVd	13								13		13
*Tomato planta macho viroid* a	TPMVd	16									15	16

^a^ For *Tomato planta macho viroid* primer design, sequences of *Mexican papita viroid* were included to give a total of 16 sequences for this conspecific species.

^b^ For analysis, only full-length sequences available on GenBank for each species at the time of array design (May 2011) were included.

^c^ Acronyms refer to assays of the multiplexed array, specific for: CSVd - *Chrysanthemum stunt viroid*; CEVd - *Citrus exocortis viroid*; CLVd - *Columnea latent viroid*; IrVd-1 - *Iresine viroid 1*; PCFVd - *Pepper chat fruit viroid*; PSTVd - *Potato spindle tuber viroid*; TASVd - *Tomato apical stunt viroid*; TCDVd - *Tomato chlorotic dwarf viroid*; TPMVd - *Tomato planta macho viroid*; PospUni - pospiviroid universal assay.

^d^ Predictions based on the maximum number of mismatches of TSPE primers n = 1. No mismatches were allowed in the final three positions of the 3′ end of TSPE primer for a given assay.

**Figure 1:**
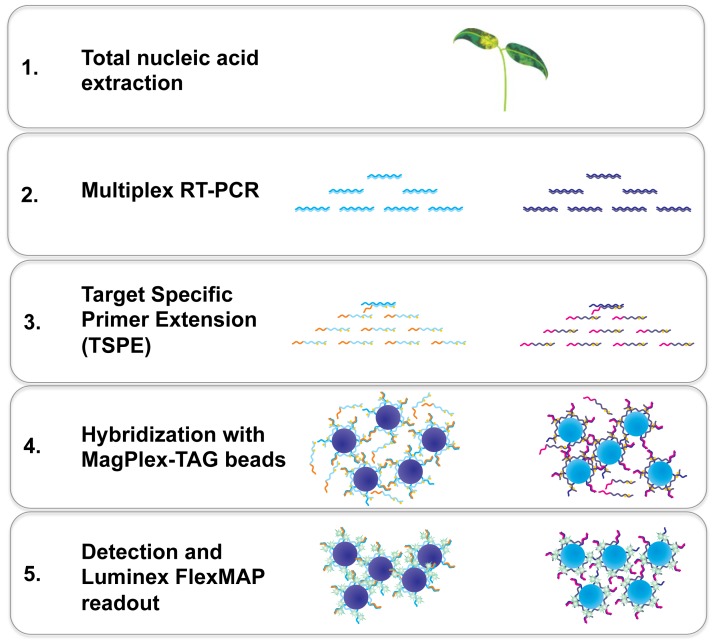
Flow diagram depicting the five steps of the Luminex MagPlex-TAG pospiviroid array method.

The final optimized conditions of the multiplexed array are detailed in Figure 1. Following total nucleic acid extraction of plant samples, a multiplexed RT-PCR step was used which utilizes seven degenerate primers to amplify a ∼270 nt region of the pospiviroid genome, and also co-amplify an ∼180 nt region of the NADH dehydrogenase plant mRNA gene. During preliminary optimization experiments, the optimal annealing temperature and cycling conditions were determined by thermal gradient PCR. The final conditions were shown to produce specific and robust amplicons of the expected sizes (for viroid and host plant targets), verified by 2.0% agarose gel electrophoresis (data not shown). A post-PCR purification step was then performed utilizing size-exclusion PCR filter plates to remove excess primers and unincorporated dNTPs. This treatment was shown to give equivalent results to the more commonly used enzymatic treatment methods (e.g. ExoSAP-IT, Affymetrix, Santa Clara, CA), which were found to be more time-consuming and costly (data not shown). The next step of the multiplex array involved a highly specific multiplexed target specific primer extension step using a mix of 11 TSPE primers, during which biotin-dCTP was incorporated into the extension products. The TSPE primers are chimeric, containing both viroid (or plant) specific sequence and a unique TAG sequence appended to the 5′ end of the primer. Following TSPE, single-stranded biotinylated extension products were hybridized to a mixture of 11 types of optically distinct Luminex MagPlex-TAG beads, each displaying a single type of anti-TAG sequence on its surface that is complementary to a TAG sequence appended to a TSPE primer. The mixture was then washed, and streptavidin-R-phycoerythrin (SAPE) added, which bound to the incorporated biotin-dCTP. The TSPE products/MagPlex bead/SAPE complexes were then sorted and detected using a Luminex FlexMAP 3D instrument.

### Specificity of the Multiplexed Bead-based Array

**Figure 2:**
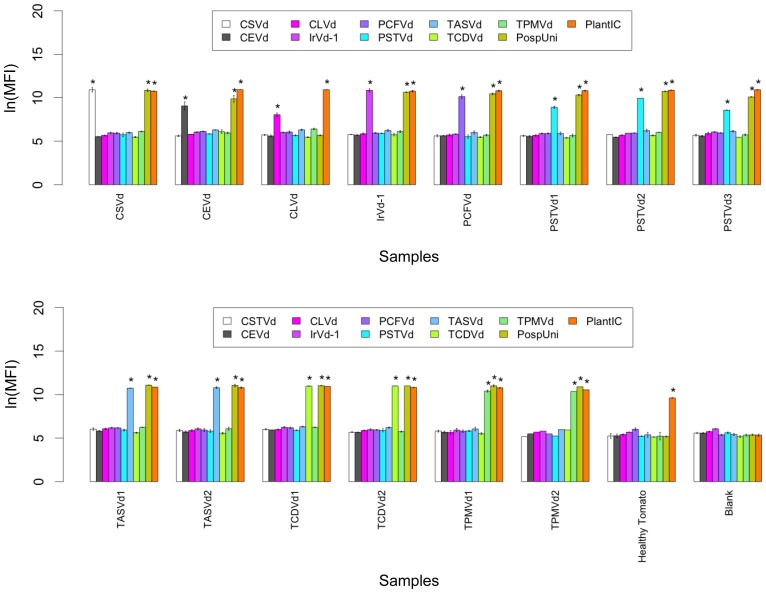
Specificity of the Luminex MagPlex-TAG pospiviroid array when screened against sequence-characterized pospiviroid isolates. Performance of the 11-plex bead-based array when screened against a large panel of single and mixed infections of sequence-characterized pospiviroid isolates, obtained from natural infections of a variety of host plants (with mixed infections simulated). Data for healthy tomato (uninfected control) and blank (no template control) samples are included for reference. Asterisks denote mean natural log (ln) median fluorescence intensity (MFI) values that exceed the threshold for that assay within the multiplexed bead-based array.

The specificity of the array was tested against a panel of 14 sequence-characterized isolates representing all nine currently recognized pospiviroid species. The multiplexed array correctly identified all pospiviroid isolates with 100% accuracy, with MFI values exceeding threshold values for the correct species-specific assays and for the PospUni assay (except for the CLVd isolate, as expected), whilst co-amplifying the plant internal control assay (Figure 2). Blank controls showed no significant increase in MFI for any of the assays and healthy tomato controls demonstrated a positive signal for the plant internal control assay (PlantIC) only.

### Sensitivity and Reproducibility of the Multiplexed Bead-based Array

In order to determine the limit of detection (LOD) of the array, ten-fold serial dilutions of RNA extracts from PSTVd isolate #N were tested using the multiplexed array. The LOD for the PospUni assay was determined to be 26.8 pg/µL of total input genomic RNA. The LOD for the PSTVd-specific assay was determined to be 2.68 ng/µL total input genomic RNA (Table 2). The plant internal control assay showed positive readings exceeding the threshold of positivity for all dilutions.

**Table 2:** The sensitivity of the multiplexed Luminex MagPlex-TAG pospiviroid array when tested using a ten-fold serial dilutions of a total RNA extract from a *Solanum lycopersicum* sample infected with *Potato spindle tuber viroid* (PSTVd, isolate #N).

PSTVd RNAconcentration (g/µL)^a^	Assays of the Luminex MagPlex-TAG Pospiviroid array (mean MFI ± standard deviation)b,c,d										
	CSVd	CEVd	CLVd	IrVd-1	PCFVd	PSTVd	TASVd	TCDVd	TPMVd	PospUni	PlantIC
2.68E-07	411±17	300±6	373±22	446±6	408±88	**7651±517**	602±80	328±15	494±11	**45330±260**	**57393±1277**
2.68E-08	295±99	273±64	290±7	466±10	411±7	**6244±227**	517±28	241±41	443±0	**49506±1936**	**53159±1619**
2.68E-09	255±22	278±57	276±1	311±16	380±14	**1179±93**	330±128	193±1	256±0	**30707±179**	**43386±244**
2.68E-10	191±14	201±26	231±24	277±42	317±34	457±48	246±50	182±11	254±15	**19679±415**	**22976±1187**
2.68E-11	180±6	170±48	205±2	300±33	388±33	236±51	285±66	142±1	188±30	**1899±186**	**19846±1155**
2.68E-12	145±13	190±17	175±13	248±6	390±52	146±60	213±33	177±17	179±26	367±14	**13226±126**
2.68E-13	182±74	202±32	255±5	293±30	367±14	197±11	184±62	159±11	210±14	247±25	**13995±1152**

^a^ Each sample was tested in triplicate.

^b^ Acronyms refer to assays of the multiplexed array specific for: CSVd - *Chrysanthemum stunt viroid*; CEVd - *Citrus exocortis viroid*; CLVd - *Columnea latent viroid*; IrVd-1 - *Iresine viroid 1*; PCFVd - *Pepper chat fruit viroid*; PSTVd - *Potato spindle tuber viroid*; TASVd - *Tomato apical stunt viroid*; TCDVd - *Tomato chlorotic dwarf viroid*; TPMVd - *Tomato planta macho viroid*; PospUni - pospiviroid universal assay; PlantIC - plant internal control assay.

^c^ Mean median fluorescence intensity (MFI) values (± standard deviation) are presented for each test in the array.

^d^ Results in bold denote values that exceed the threshold for positivity, whereby thresholds were calculated using the novel non-parametric threshold setting method described in this study.

**Figure 3:**
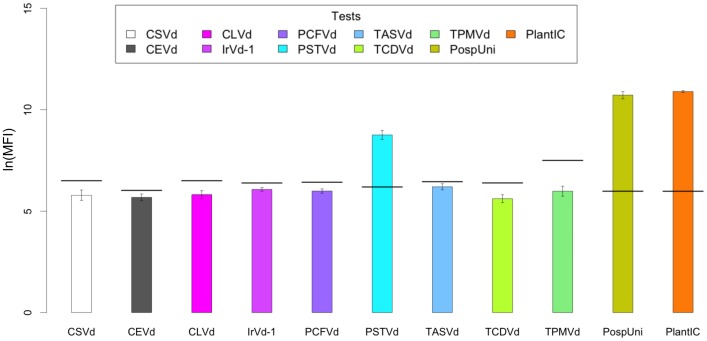
Reproducibility of the Luminex MagPlex-TAG pospiviroid array over ten independent tests. One sample of *Potato spindle tuber viroid* (isolate #3077695) was tested in ten independent reactions over several days. Mean natural log (ln) median fluorescence intensity (MFI) values are plotted; error bars show plus or minus (±) one standard deviation. The horizontal bars plotted on each of the bars shows the detection threshold obtained from our kernel density estimation method, for each assay of the array. The small standard deviation of the ln(MFI) values for positively reacting assays in the array (PSTVd, PospUni and PlantIC) demonstrate the high level of precision of the multiplexed bead-based array.

The inter-assay reproducibility of the array was tested by ten independent trials with PSTVd isolate #3077695. One sample *t*-tests showed that only samples for which the assays were specific had significantly higher average natural log (ln) MFI values than the thresholds (*p*<0.05). Importantly, the standard deviations of ln(MFI) values for each of the assays were small (Figure 3), demonstrating the high precision of the multiplexed array.

### Blind Testing of Single and Mixed Infections

A total of 11 plant nucleic acid extracts containing single and simulated mixed infections of pospiviroids (including two healthy tomato controls) were correctly identified by the multiplexed array in a blind setting, when tested by an independent operator at an independent facility (Table 3). There was one discrepancy in the results for the CSVd sample, which gave positive results for the CSVd and PlantIC assay of the array, but failed to be identified as positive in the PospUni assay as expected.

**Table 3:** Results of the blind testing of single and mixed infections of pospiviroids in plant samples, showing a comparison of three separate methods for setting thresholds for positivity.

	Assays of the Luminex MagPlex-TAG pospiviroid arraya,b,c										
Samples	CSVd	CEVd	CLVd	IrVd-1	PCFVd	PSTVd	TASVd	TCDVd	TPMVd	PospUni	PlantIC
CSVd	415**	55	77	264	161	100	623	247	108	1001	10057***
CEVd +TCDVd	172	42541***	186	390	526	171	2038	6236***	380	52636***	25679***
CLVd +TCDVd	158	132	2328**	1013	1081	142	4707*	578**	680	12640***	58345***
IrVd-1+ PSTVd+TCDVd	133	121	187	28821***	427	489**	1783	423**	409	24079***	14937***
PCFVd	88	61	245	562	2425**	150	3311*	172	340	2372**	40987***
PSTVd	193	93	198	287	467	7794***	1625	288	191	16183***	23642***
TASVd	177	123	391	520	476	209	22959***	172	239	21317***	23596***
TCDVd	109	62	216	457	404	97	2736	445**	342	2970**	29558***
TPMVd+PSTVd	149	85	180	409	339	636**	1366	87	7759***	17403***	8101***
Healthy tomato	143	112	243	668	687	155	2965	207	370	845	44083***
Healthy tomato	111	159	349	792	879	76	5250*	206	409	903	61464***
No template control	69	48	37	168	106	65	80	49	67	23	26

^a^ Mean median fluorescence intensity (MFI) values are presented for each assay of the array.

^b^ Acronyms refer to assays of the multiplexed array specific for: CSVd - *Chrysanthemum stunt viroid*; CEVd - *Citrus exocortis viroid*; CLVd - *Columnea latent viroid*; IrVd-1 - *Iresine viroid 1*; PCFVd - *Pepper chat fruit viroid*; PSTVd - *Potato spindle tuber viroid*; TASVd - *Tomato apical stunt viroid*; TCDVd - *Tomato chlorotic dwarf viroid*; TPMVd - *Tomato planta macho viroid*; PospUni - pospiviroid universal assay; PlantIC - plant internal control assay.

^c^ Asterisks indicate values that exceed the threshold for positivity using one or more of the three threshold setting methods compared in this analysis. Method 1 utilizes the novel, non-parametric data-driven threshold setting method described in this study. Method 2 utilizes the arbitrary threshold cut-off set at the value of the mean MFI plus two-fold standard deviation of healthy (uninfected) control samples. Method 3 utilizes the arbitrary threshold cut-off set at the value of the two times the mean MFI of healthy (uninfected) control samples, calculated separately for each assay of the array. Values with three asterisks denote positive samples correctly identified using all threshold-setting methods (Methods 1, 2 and 3). Values with two asterisks denote positive samples correctly identified using Methods 1 and 3 only (and thereby denote false negative samples using Method 2). Values with one asterisk denote false positive samples identified using Method 2.

### Identifying a Threshold for Positivity

The signal-to-noise ratios (mean divided by standard deviation of replicate MFI values) obtained from the multiplexed array measurements were usually very high (Tables 2 and 3), enabling the straightforward recognition of positive samples. However, in order to remove subjectivity and incorporate statistical rigor into the process of attributing a positive/negative result to the MFI results for each sample, we developed a nonparametric, fit-for-purpose statistical analysis method for defining thresholds for positivity for each separate pospiviroid species assay within the array (R code for this analysis is provided as supplementary material S1). A key component of the method was the estimation of the probability distribution of background MFI values for a particular viroid species assay within the array, using non-target samples where the assay was not designed to give a positive result. Importantly, we estimated this distribution using kernel density estimation on the ln(MFI) data, which freed our method from needing to make parametric assumptions about the distribution of the background MFI values (e.g. assuming a normal distribution). We then selected the 99^th^ percentile of this distribution as the threshold for positivity, so that there was only a 1% chance of a false positive (Type I error), when a sample was randomly sampled from the background MFI distribution. To impose a further tier of rigor, where replicate MFI values were obtained for a particular assay, a positive result was only declared if the mean MFI was deemed to be significantly greater than the threshold of positivity at the 0.01 significance level with a one sample *t*-test.

The thresholds for positivity in our study were calculated upon a predefined acceptable risk for falsely attributing a positive result to a non-target sample (e.g. we used 1%). Whilst we chose the 99^th^ percentile for the analyses, there is no reason that the method could not be used with a slightly lower or higher quantile. Choice of a quantile is a subjective assessment and dependent on the level of risk the diagnostician is willing to take for making Type I errors. Choosing a lower quantile will reduce the threshold, but increase the probability of a false positive. Using our methodology, one can easily relate a given threshold to the probability of a Type I error.

A comparative analysis was performed to assess the efficacy of the nonparametric threshold setting method described in this study with two alternative arbitrary methods. The nonparametric threshold setting method (Method 1) was compared with setting the threshold cut-off at the value of the mean MFI plus two-fold standard deviation of healthy (uninfected) control samples (Method 2). Method 1 was also compared with setting the threshold cut-off at two times the mean MFI of healthy (uninfected) control samples, calculated separately for each assay of the array (Method 3). When the results of the multiplexed array specificity validation were analyzed using the three threshold setting methods (Table 4), Method 1 resulted in no false positive and negative results (0%), Method 2 resulted in high overall false positive rates for raw MFI data (81%) and ln(MFI) data (88%), and Method 3 resulted in high overall false positive (63%) and false negative (88%) for the raw MFI data and ln(MFI) data, respectively. When the results of the blind testing of pospiviroid samples were assessed using the three threshold setting methods (Table 3), Method 1 again resulted in no false positives and negatives, as did Method 3. Method 2 however resulted in an overall false positive rate of 27% (values with one asterisk in Table 3) and false negative rate of 55% (values with two asterisks in Table 3) for raw MFI data.

**Table 4:** Percentages of false positive and false negative results for Luminex MagPlex-TAG pospiviroid array data when analyzed using three different methods for setting thresholds for positivity.

	Analysis of Luminex MagPlex-TAG pospiviroid array data^a^					
	ln(MFI)			MFI		
	Method 1^c^	Method 2^d^	Method 3^e^	Method 1^c^	Method 2^d^	Method 3^e^
**False positives** b	0	88	0	0	81	63
**False negatives** b	0	0	88	0	0	0

^a^ The Luminex MagPlex-TAG pospiviroid array results for 14 sequence-characterized pospiviroid samples (in addition to negative no template control and healthy uninfected control samples) analyzed by three threshold setting methods, using both raw MFI and natural log (ln) transformed MFI data.

^b^ The percentage of false positive/negative for each method were calculated by scoring each sample with an array result, whereby an overall score for that sample was recorded as a false positive/negative if a false positive/negative result was called for at least one assay in the array.

^c^ Method 1 utilizes the novel, non-parametric data-driven threshold setting method described in this study.

^d^ Method 2 utilizes an arbitrary threshold cut-off set at the value of the mean MFI plus two-fold standard deviation of healthy (uninfected) control samples for all assays within the array.

^e^ Method 3 utilizes an arbitrary threshold cut-off set at the value of two times the mean MFI of healthy (uninfected) control samples for a given assay within the array.

## Discussion

The ability to quickly and accurately screen planting material (both symptomatic and asymptomatic) and seeds for the presence of pospiviroids is critical to preventing their introduction and spread in both glasshouse and field crops. This study reports the development and validation of the 11-plex Luminex MagPlex-TAG Pospiviroid array, a valuable tool for the simultaneous detection of all nine recognized viroid species in the genus *Pospiviroid*, from both crop and ornamental plant species. To our knowledge, this is the first report of Luminex nucleic acid-based detection of pospiviroids, and also the first true multiplexed method for the single-assay detection of all nine recognized pospiviroid species.

The array has a unique hierarchical design, incorporating a near-universal assay that detects all pospiviroid species (except for the sequence divergent CLVd), in addition to species-specific assays that detect CSVd, CEVd, CLVd, IrVd-1, PCFVd, PSTVd, TASVd, TCDVd, and TPMVd (including isolates previously characterized as MPVd). An internal control assay designed to co-amplify plant mRNA (NADH dehydrogenase subunit 5 gene) is also included to exclude false negative results. The near-universal assay (PospUni), designed to the upper central conserved region of the pospiviroid genome, was incorporated into the array design as a secondary internal control to provide an amplification signal for all pospiviroid species. Based on currently available sequence data, the multiplexed array described here should correctly identify the majority of isolates of each species of pospiviroid.

The overall design of assays within the array was constrained by the very small genome size and the frequency of highly repetitive nucleotide motifs (a feature that contributes to the highly base-paired secondary structure of the native viroid genomic RNA). Also, the level of multiplexing in this array required seven oligonucleotide primers to be present in each single multiplexed PCR, creating the potential for non-specific interactions. All of these factors were taken into account for the *in silico* design of the assays within the array. In practice, the array demonstrated 100% specificity when evaluated against a large panel of sequence-characterized viroid-infected plant samples. Mixed infections were accurately identified, proving the multiplex capability to identify pospiviroids in samples containing single as well as mixed infections. The PlantIC internal control assay was shown to co-amplify plant RNA from all plant species tested, including *S. lycopersicum*, *S. tuberosum*, *Argyranthemum* spp., *Celosia* spp., *Brugmansia* spp. and *Petunia* spp. Because the PlantIC assay co-amplifies host RNA, it enables the monitoring of the entire diagnostic process from initial RNA extraction to diagnostic result. The PlantIC assay was incorporated for quality assurance purposes, to exclude possible false negative results that can occur due to RNA degradation, human error and PCR inhibition caused by co-extracted impurities.

The sensitivity of the multiplexed array was examined by testing a dilution series of PSTVd-infected RNA extracts. The multiplexed array reliably detected PSTVd samples diluted up to 100 times, with positive signals exceeding the thresholds for the PSTVd, PospUni and PlantIC assays in the array (as expected). This level of sensitivity is suitable for the diagnosis of naturally infected samples, and is likely a result of the highly multiplexed nature of the detection technology and the degeneracy of primers used for the multiplexed amplification step. Moreover, this level of sensitivity may reduce the risk of false positive results, a factor that has been previously highlighted as a serious risk for other ultra-sensitive real-time PCR detection assays for pospiviroids [21], [28]. The array was tested for reliability by assaying one PSTVd sample independently ten times over separate days. The ln(MFI) values consistently showed low variability and only the assays for which the sample was positive were statistically significant. This demonstrates the high level of reproducibility of the array, even when performed on separate days.

The performance of the multiplex array was validated successfully under blind testing conditions, demonstrating the robust nature of this diagnostic technology. The only exception was the CSVd sample, which gave positive signals for the CSVd and PlantIC tests of the array, but did not produce a positive signal for the PospUni test as expected. This result was hypothesized to have occurred due to a low concentration of viroid target in the plant extract, indicated by the contrast between the relatively high MFI result for the PlantIC test (MFI 10057) with the low MFI result for the CSVd test (MFI 415).

To determine if samples were positive or negative for a given pospiviroid in the array, a new statistically driven method for determining the threshold was developed. For our purposes (i.e. true multiplex detection), a statistically rigorous method was needed to ensure that the results were objective and easily reproduced in other laboratories. To date, no data-driven, statistical methods have been described for determining detection thresholds for Luminex multiplexed bead-based arrays. The method we describe provides a clear and unambiguous approach to defining thresholds for positivity for each individual assay within the same array, a feature that allows the unique interactions and properties of the assays within the array to be adequately accounted for. The applicability of the method is not limited to assays of the Luminex multiplexed array format, and could be used to predict cut-off threshold values for other simplex or multiplexed quantitative assay formats, e.g. enzyme-linked immunosorbent assay.

The threshold setting method we describe here was shown to perform better than alternative arbitrary methods commonly used for serodiagnostic assays [40]–[42]. Arbitrarily defining the cut-off threshold of an assay as either (i) the mean plus two-fold standard deviation of the result of healthy (uninfected) control samples, or (ii) two times the mean MFI of negative healthy (uninfected) control samples calculated separately for each assay of the array, resulted in very significant proportions of overall false positives or false negatives. A sample was considered to have an “overall” false positive or negative result if one or more of the individual pospiviroid species assay in the multiplexed array gave a false positive or negative result for that sample. These alternative arbitrary threshold-setting methods do not account for the possible interactions that may occur between the mixture of different primers and targets. These interactions could cause elevated background levels resulting in false positives when results are analyzed using conventional arbitrarily defined threshold setting methods.

This study presents a new multiplexed platform for the single-assay detection of pospiviroids. The multiplexed array is well suited for upscaling for use in high-throughput detection laboratories. It offers a number of advantages over other systems currently used for the diagnosis of pospiviroid diseases. This array has the ability to simultaneously detect, in one single assay, all nine species in the genus *Pospiviroid*, including the sequence-divergent CLVd, without the need for an additional verification step. The presence of the generic pospiviroid marker PospUni not only adds an internal control but also an extra layer of reliability to the assay since the presence of one or more pospiviroids in a sample should result in a minimum of two positive signals. A co-amplified internal control was incorporated into the assay for quality assurance purposes, while the assay format makes it suitable for utilizing liquid handling robotics. The development of the R software driven data analysis tool allows for unambiguous and easy interpretation of assay results. An important feature of the multiplexed array is the flexibility to add or subtract assays as required. This aspect that may prove to be immensely important in the future if new variants, strains and species are discovered. Overall, the results demonstrated that the array was easy to perform, specific and sensitive to the target sample being tested, and gave reproducible results in multiple assays. The multiplexed bead-based array described in this study has a strong potential for application in pospiviroid surveillance and diagnostics, to facilitate improvements in pospiviroid disease management.

## Materials and Methods

### Plant Samples and Nucleic Acid Extractions

Leaves of viroid-infected plants used for assay development and validation were kindly provided by M. Botermans (Dutch Plant Protection Service, Wageningen, The Netherlands) and stored at −20°C (Table 5). Total RNA was extracted from 100 mg of frozen leaf material using the RNeasy Plant Mini Kit (QIAGEN, Valencia, CA) according to the manufacturer’s protocol, and stored at −20°C. In order to simulate a plant infected with more than one viroid species, total RNA extracts from separately infected plants were mixed prior to testing.

**Table 5:** Details of pospiviroid isolates used for validation of the Luminex MagPlex-TAG pospiviroid array.

Viroid species	Acronym	Matrix (host plant)	Isolate code	GenBank accession^b^
*Chrysanthemum stunt viroid*	CSVd	*Argyranthemum spp*.	4783858	N/A
*Citrus exocortis viroid*	CEVd	*Solanum tuberosum*	3823889	EU094208
*Columnea latent viroid*	CLVd	*S. tuberosum*	93007481	AY372392
*Iresine viroid 1*	IrVd-1	*Celosia spp.*	4416011	GU911350
*Pepper chat fruit viroid*	PCFVd	*S. tuberosum*	3259237	FJ409044
*Potato spindle tuber viroid*	PSTVd1	*S. tuberosum*	3077695	EF192393
*Potato spindle tuber viroid*	PSTVd2	*Solanum lycopersicum*	N	X17268
*Potato spindle tuber viroid*	PSTVd3	*S. tuberosum*	Howell	AY372400
*Tomato apical stunt viroid*	TASVd1	*S. tuberosum*	3153272	N/A
*Tomato apical stunt viroid*	TASVd2	*S. lycopersicum*	3153272	N/A
*Tomato chlorotic dwarf viroid*	TCDVd1	*Brugmansia spp.*	3816013	EF626530
*Tomato chlorotic dwarf viroid*	TCDVd2	*Petunia spp.*	Q06383	GQ396664
*Tomato planta macho viroid*	TPMVd1	*S. lycopersicum*	3289954	K00817
*Tomato planta macho viroid* a	TPMVd2	*S. lycopersicum*	OG1	L78454

^a^ Formerly classified as *Mexican papita viroid* (MPVd).

^b^ N/A indicates not available.

### Multiplexed Array Design

The overall scheme of the multiplexed array is shown in Figure 1. For the design of pospiviroid species-specific and universal assays, all 642 available (May 2011) full-length pospiviroid sequences were retrieved from the National Center for Biotechnology Information (NCBI) GenBank database. Sequences were aligned using ClustalW [43], implemented in Geneious version 5.4.3 (Biomatters Ltd, Auckland, New Zealand). Based on these multiple sequence alignments, candidate regions were identified that best demonstrated (i) sequence conservation within a species whilst exhibiting sequence divergence between species for the design of species-specific assays, and (ii) sequence conservation across all species for the design of the universal assay, excluding the sequence divergent CLVd species. For reference, *in silico* analysis was performed to predict the number of isolates likely to be detected with each assay (Table 1). Multiplex RT-PCR primers and target specific primer extension (TSPE) primers were designed to these regions according to established guidelines for real-time PCR assays [44], with primer characteristics checked using Beacon Designer Free Online Tools (Premier Biosoft International, Palo Alto, CA) and specificity checked against sequence data available on GenBank using BLAST (NCBI). The plant internal control assay (PlantIC), specific for mRNA of the mitochondrial NADH dehydrogenase subunit 5 (*nad5*) gene, was adapted from a real-time PCR assay described by Botermans et al. [29]. Each TSPE primer was designed with a unique MagPlex-TAG sequence appended to the 5′ end, complementary to the anti- MagPlex-TAGs displayed on the surface of the corresponding MagPlex-TAG bead address (Luminex Corporation, Austin, USA). Final primers are listed in Table 6 and were synthesized by Biolegio (Nijmegen, The Netherlands).

**Table 6:** Characteristics of the oligonucleotide primers used in this study.

General assay target	Multiplex PCR primers (5′-3′)a, b		Amplicon size^c^	TSPE primer sequences (5′-3′)d, e		Target	MagPlex-TAG bead address^f^
Pospiviroid	PospF1	CC**W**GTGGT**K**C**W**C**W**CCTGACC	∼270 nt	tPospUni	CTAAATCACATACTTAACAACAAA GGGATCCCCGGGGAAACCTGGA	All Pospiviroidspecies (-CLVd)	63
	PospF2	GCACCCCTGACCTGCAATG		tCLVd	TTAATACAATTCTCTCTTTCTCTA AGAGCGCAAGAGCGGTCTC	CLVd	54
	PospF3	GGTGCCTGTGGTGCCTC		tCSVd	ATACTTTACAAACAAATAACACAC AGGAAGTCCGACGAGATCGC	CSVd	19
	PospR1	CAGTTGT**WK**CCACCGGGTAGT		tCEVd	ACAAATATCTAACTACTATCACAA CGGATCACTGGCGTCCAGC	CEVd	39
	PospR2	CCACCGGTCGCGTCAG		tIrVd	CAAACAAACATTCAAATATCAATC GTCGACCGCGTAAAGACCGGA	IrVd-1	22
				tPSTVd	TTAACAACTTATACAAACACAAAC GCGGCCGACAGGAGTAATTCC	PSTVd	53
				tTCDVd	AATCAACACACAATAACATTCATA GCAAAAGGCGGCAGGGAGC	TCDVd	48
				tTASVd	AATTTCTTCTCTTTCTTTCACAAT CGAGGTCGGGGGCTTCGGA	TASVd	14
				tPCFVd	CTATCATTTATCTCTTTCTCAATT GAAAGGGGAAGCAAGCATCTCCT	PCFVd	72
				tTPMVd	TCATCACTTTCTTTACTTTACATT GTCGCGGCTGGGGAGTCTC	TPMVd^e^	44
Plant Internal control (PlantIC)	Nad5F	GATGCTTCTTGGGGCTTCTTGTT	181 nt	tNad5	AACTTTCTCTCTCTATTCTTATTT AGGATCCGCATAGCCCTCGATTTATGTG	Plant mRNA NADHgene	43
	Nad5R	CTCCAGTCACCAACATTGGCATAA					

^a^ The nucleotides in bold are degenerate sites, with ambiguity codes as follows: W = A or T; K = G or T.

^b^ F, R and t represent forward primer, reverse primer and target specific primer extension (TSPE) primer, respectively.

^c^ Amplicon size differs between species of pospiviroids due to differences in length of genomes (insertions and deletions over amplification region).

^d^ Underlined segment of primer indicates the MagPlex-TAG sequences provided by Luminex, which bind to the complementary anti-MagPlex-TAG sequenced displayed on the surface of the corresponding bead address.

^e^ For *Tomato planta macho viroid*, the target of detection includes isolates of *Mexican papita viroid*, as this viroid is considered a conspecific.

^f^ The MagPlex-bead product number assigned by Luminex (Luminex Corp., Austin, TX, USA).

### Multiplex Two-step RT-PCR and Post-PCR Purification

To initiate cDNA synthesis, a mixture containing 2 µL of RNA extract, 300 nM each of the reverse primers PospR1, PospR2 and Nad5R (Table 6) and nuclease-free distilled water (dH_2_0) to a final volume of 10 µL was incubated at 80°C for 10 min and snap-cooled on ice. The cDNA synthesis reverse transcription (RT) reaction mixture was then added, which contained 4 µL of 5×first strand buffer (Invitrogen, Carlsbad, CA, USA), 10 mM dithiothreitol (Invitrogen), 0.5 mM dNTPs (Invitrogen), 10 U RNAse OUT (Invitrogen), 50 U Superscript III (Invitrogen), and dH_2_0 to a final volume of 10 µL, and incubated at 50°C for 45 min, then 70°C for 15 min. Following cDNA synthesis, multiplexed PCR was done using seven PCR primers PospF1, PospF2, PospF3, PospR1, PospR2, Nad5F, Nad5R (Table 6), which were designed to amplify all pospiviroid sequences and the plant mRNA internal control. Each 25 µL reaction contained 3 µL cDNA template, 1 U Platinum *Taq* DNA Polymerase (Invitrogen), 250 nM of each PCR primer, 100 µM of each dNTP (Invitrogen), 1.5 mM MgCl_2_ and 1×PCR buffer (supplied). Cycling conditions for the multiplex PCR were 94°C for 1 min, 30 cycles of 94°C for 30 s, 58°C for 30 s, and 72°C for 30 s, with a final extension of 72°C for 3 min. All PCRs were performed in 96 well plates using a Veriti thermal cycler (Applied Biosystems, Foster City, CA, USA). Amplicons were purified using Montage PCR clean-up filter plates (Millipore, Schwalbach, Germany) to remove excess primers and dNTPs. Purified amplicons were used as templates for target-specific primer extension (TSPE) reactions utilizing target specific MagPlex-TAG primers. During initial experiments, multiplexed RT-PCR products were verified by 2% agarose gel electrophoresis with GelRed (Biotium, Hayward, CA, USA) staining.

### Multiplex TSPE

Linear TSPE reactions contained 5 µL of purified multiplex PCR amplicons, 0.75 U Platinum GenoTYPE *Tsp* DNA polymerase (Invitrogen), 25 nM of each of the 11 TSPE primers (Table 6), 5 µM each of dATP, dGTP, dTTP and biotin-dCTP (Invitrogen), 1.25 mM MgCl_2_, 1×PCR buffer (Invitrogen). Cycling conditions were 96°C for 2 min, 30 cycles of 94°C for 30 s, 58°C for 1 min, and 74°C for 1 min.

### Hybridization and Detection

Microsphere hybridization reactions were performed according to the manufacturer’s recommendations (Luminex Corp., Austin, TX, USA), with minor modifications. All washing steps were performed using a magnetic plate separator (Luminex). To prepare the mixture of 11 optically distinct Luminex MagPlex-TAG beads (Table 6), each bottle of MagPlex-TAG microspheres was vortexed vigorously for 1 min, then sonicated for 30 s. Bead mixtures were prepared in Eppendorf LoBind tubes (Eppendorf, Hamburg, Germany), diluted with 2×Tm hybridization buffer (0.2 M Tris-HCl pH 8.0, 0.4 M NaCl, 0.16% Triton X-100) to yield a final concentration of approximately 1250 beads of each bead address per 25 µL. The bead mixture was vortexed again for 30 s, then aliquoted in 25 µL portions into 96-well skirted microplates (Greiner Bio-One, Kremsmünster, Germany). Single-stranded biotinylated linear amplicons (5 µL) were added per well, with the total reaction volume adjusted to 50 µL per reaction with dH_2_0. For negative (no template) control wells, 25 µL of dH_2_0 was added to the bead mixture. Reactions were denatured at 96°C for 60 s, then hybridized at 37°C for 30 min. After hybridization, reactions were shaken for 5 min using an IKA MS1 microplate shaker (IKA®-Werke GmbH, Staufen, Germany), then washed twice in 75 µL of 1×Tm Buffer and resuspended in 50 µL of 1×Tm Buffer containing 2 µg/mL streptavidin-*R*-phycoerythrin conjugate (SAPE; Prozyme, San Leandro, CA, USA). Plates were incubated at 37°C for 15 min, then washed once in 75 µL of 1×Tm Buffer, resuspended in 75 µL of 1×Tm Buffer and shaken for 5 min. Samples were analyzed and microsphere complexes detected using a Luminex FlexMAP 3D instrument (Luminex). The MFI values for biotinylated extension products attached to 100 microspheres of each individual bead address were measured for each assay, and each sample tested in triplicate. Data are shown as raw MFI ± SD unless otherwise indicated. The average MFI from three template-free control samples was also determined to monitor background fluorescence, and background MFI subtraction was not used.

### Sensitivity and Inter-assay Reproducibility

To determine the lower limit of detection (LOD) of the multiplexed array, six ten-fold serial dilutions of total RNA extracted from a naturally infected PSTVd isolate #N were prepared, ranging from 268 ng/µL to 268 pg/µL in a background of healthy tomato RNA (1/500 dilution of neat extract), and tested in triplicate. Total RNA concentrations were quantified by spectrophotometry using an Eppendorf Biophotometer (Eppendorf, Hamburg, Germany). To evaluate the inter-assay reproducibility of the array, one neat extract of PSTVd isolate #3077695 was tested in ten independent reactions over separate days, with mean ln(MFI) values and error bars showing the standard deviations plotted in Figure 3. The variability of the ln(MFI) values was assessed by one sample *t*-tests, with *p*-values <0.05 considered statistically significant.

### Blind Testing of Single and Mixed Infections

To further assess the performance of the Luminex MagPlex-TAG Pospiviroid array, 11 “blind” samples of unknown identity were tested by an independent operator at an independent facility (Naktuinbouw, Roelofarendsveen). The sample panel included both single and simulated mixed infections, in addition to healthy tomato controls. All blind samples were previously characterized using DNA sequence analysis (data not shown) and were provided to the operator as total nucleic acid extracts. Samples were tested using the multiplexed array according to the conditions described in this study, except that microsphere hybridization reactions were detected using a Luminex MAGPIX instrument (Luminex).

### Determination of Thresholds for Positivity

A non-parametric, data-driven statistical method for setting thresholds for positivity was developed. Thresholds were set for each individual assay to define whether a sample was positive or negative. For a given assay, the natural log (ln) of MFI values from non-target samples (for which the assay should be negative) were used to estimate a distribution, herein referred to as the null distribution for that assay. Samples representing the full diversity of pospiviroid species were used to generate this distribution. The null distribution was estimated using kernel density estimation and the threshold for positivity was then taken as the 99^th^ percentile of this. In a scenario where more than 100 non-target ln(MFI) values were available, it would be possible to identify the 99^th^ percentile from the empirical distribution function of the data. However, in practice only a relatively small number of samples are available to estimate the null distribution, hence our reliance on kernel based methods.

The statistical procedure was as follows: for any given assay, *A*, let *Y_1_, Y_2_, …, Y_m_* and *X_1_, X_2_, …, X_n_* denote ln(MFI) values obtained for a selection of samples for which the assay is specific and non-specific, respectively. Using the *X_i_* values, the probability density function of background ln(MFI) was estimated using a kernel density estimator 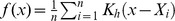, where 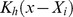 is a Gaussian kernel function, with bandwidth *h*. The density and bandwidth were computed using the “density” function in the R statistical language [45]. The detection threshold, 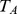, for assay *A* was then defined as the 99^th^ percentile of 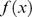. If multiple runs were available for the analysis, then an assay was deemed to be specific for the target sample if 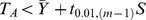, where 
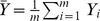
, 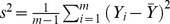
and 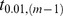 is the 1^st^ percentile of the t-distribution with *(m-1)* degrees of freedom. If only a single run was available for the analysis, then an assay was deemed to be specific for the target sample if 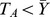. Clearly, the percentiles of 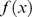 can be easily adjusted depending on the practitioner’s tolerance for statistical Type I error. The annotated R code for this procedure is presented in the supplementary material file S1.

To evaluate the efficacy of the nonparametric threshold setting method described in this study, a comparative analysis was performed using two additional, commonly used arbitrary threshold setting methods. Data were analyzed firstly by using the nonparametric method we describe in this study (Method 1), secondly by setting the threshold cut-off at the value of the mean MFI plus two-fold standard deviation of healthy (uninfected) control samples (Method 2), and thirdly by setting the threshold cut-off at two times the mean MFI of healthy (uninfected) control samples, calculated separately for each assay of the array (Method 3). Analyses were performed on two data sets generated as part of this study: (a) the results of the specificity validation experiment, and (b) the results of the blind testing experiment where nine single and simulated mixed pospiviroid infected samples. Both experiments included appropriate healthy (uninfected) and negative (no template) control samples. Using each of the threshold setting methods, we considered a sample to have a “overall” false positive result if one or more of the target-specific assays in the multiplexed array gave a false positive result for that sample, and a “overall” false negative result if one or more of the target-specific assays in the multiplexed array gave a false negative result for that sample.

## Supporting Information

File S1 **R code with comments.** R code for determining thresholds for positivity for each separate assay in the array, using non-target (negative) data as the input values.
(TXT)
